# Individual and combined effects of deoxynivalenol (DON) with other *Fusarium* mycotoxins on rainbow trout (*Oncorhynchus mykiss*) growth performance and health

**DOI:** 10.1007/s12550-023-00496-0

**Published:** 2023-07-20

**Authors:** Paraskevi Koletsi, Geert F. Wiegertjes, Elisabeth A. M. Graat, Marijn de Kool, Philip Lyons, Johan W. Schrama

**Affiliations:** 1 Aquaculture and Fisheries Group, Wageningen University and Research, 6708 WD Wageningen, The Netherlands; 2 Adaptation Physiology Group, Wageningen University and Research, 6708 WD Wageningen, The Netherlands; 3 Alltech Biotechnology Inc, Dunboyne, A86 X006 Ireland

**Keywords:** Mycotoxins, Co-contamination, Fish, Feed, Rainbow trout, Growth

## Abstract

This study assessed whether the toxicological effects of deoxynivalenol (DON) produced by *Fusarium graminearum* in rainbow trout (*Oncorhynchus mykiss*) are altered by the co-exposure to a mixture of toxins produced by *Fusarium verticillioides* (FU_mix_). This FU_mix_ contained fusaric acid and fumonisin B_1_, B_2_ and B_3_. Four diets were formulated according to a 2 × 2 factorial design: CON-CON; CON-FU_mix_; DON-CON; and DON-FU_mix_. Diets with and without DON contained on average 2700 and 0 µg/kg feed, respectively. The sum of the analysed FU_mix_ toxins was 12,700 and 100 µg/kg feed in the diets with and without FU_mix_, respectively. The experiment consisted of a 6-week restrictive feeding period immediately followed by a 2-week *ad libitum* feeding period. Growth performance measurements were taken per feeding period. Histopathological measurements in the liver and gastrointestinal tract (pyloric caeca, midgut and hindgut) were assessed at the end of week 1 and week 6 of the restrictive feeding period and at week 8, the last day of the *ad libitum* feeding period. During both restrictive and *ad libitum* feeding, the effects of FU_mix_ and DON on growth performance were additive (no interaction effect; *p* > 0.05). During the restrictive feeding period, exposure to DON (*p* ≤ 0.001) and FU_mix_ (*p* ≤ 0.01) inhibited growth and increased feed conversion ratio (FCR). During this period, DON exposure decreased the protein (*p* ≤ 0.001) and energy retention (*p* ≤ 0.05) in the trout. During the *ad libitum* feeding period, FU_mix_ affected HSI (*p* ≤ 0.01), while DON exposure reduced feed intake (*p* ≤ 0.001) and growth (*p* ≤ 0.001) and increased FCR (*p* ≤ 0.01). In general, for both liver and intestinal tissue measurements, no interaction effects between DON and FU_mix_ were observed. In the liver, histopathological analysis revealed mild alterations, increased necrosis score by DON (*p* ≤ 0.01), increased glycogen vacuolization by FU_mix_ (*p* ≤ 0.05) and decreased percentage of pleomorphic nuclei by FU_mix_ (*p* ≤ 0.01). DON had a minor impact on the intestinal histological measurements. Over time, some of the liver (glycogen vacuolization score, pleomorphic nuclei; *p* ≤ 0.01) and intestinal measurements (mucosal fold and enterocyte width; *p* ≤ 0.01) were aggravated in fish fed the FU_mix_ contaminated diets, with the most severe alterations being noted at week 8. Overall, the co-exposure to FU_mix_ and DON gave rise to additive effects but showed no synergistic or antagonistic effects for the combination of DON with other *Fusarium* mycotoxins.

## Introduction

The diversity and inclusion level of vegetable/plant ingredients in aquafeeds have increased over the years (Turchini et al. 2019), even for carnivorous fish-like salmonids (Aas et al. 2022). This is related to multiple factors, including the continuous expansion of the aquaculture sector (Naylor et al. 2021) and thereby the increasing demand for aquafeeds (Tacon 2020), the limited availability of fishmeal and fish oil (Naylor et al. 2009) and the competition for ingredients for farmed animal feeds and biofuel production (Kraan 2013). Next to other adverse antinutritional effects, the use of grains, seeds and their by-products increases the risk of fish and shrimp being exposed to mycotoxins (Francis et al. 2001; Hardy 2010; Glencross 2016).

Mycotoxin contamination of crops by fungi can occur pre-harvest in the fields and post-harvest during transportation and storage, depending on climatic conditions (temperature and humidity) (Bryden 2012). Ongoing climate change and more extreme weather conditions affect pre-harvest fungal proliferation, which increases the risk of mycotoxin contamination (Paterson and Lima 2010; Perrone et al. 2020; Zingales et al. 2022). Aquafeeds which contain multiple plant-based ingredients can be contaminated with a mixture of different mycotoxins which are produced by one or several fungi (Streit et al. 2012; Smith et al. 2016). Indeed, surveys at the regional and country level have reported multiple mycotoxin contamination in aquafeeds (Europe, (Koletsi et al. 2021); Asia, (Gonçalves et al. 2018a); East Africa, (Marijani et al. 2017); Brazil, (Barbosa et al. 2013); Argentina, (Greco et al. 2015); Serbia, (Rokvić et al. 2020); Kenya, (Mwihia et al. 2020)). For instance, in European aquafeeds, 75% of the samples analysed were contaminated with two or more mycotoxins (Koletsi et al. 2021). In this review study, the most prevalent toxins in aquafeeds were identified as *Fusarium-*produced mycotoxins: fusaric acid (55%), deoxynivalenol (DON) (48%), fumonisin B_1_ (FB_1_) (36%) and fumonisin B_2_ (FB_2_) (27%). Fumonisins (FB_1_, FB_2_ and FB_3_) and fusaric acid are produced (often as a mixture) by *Fusarium verticillioides* and DON by *Fusarium graminearum* (Thrane 2014). *F. verticillioides* and *F. graminearum* grow under similar climate conditions in the field (Thrane 2014). Consequently the occurrence of DON often goes together with the presence of a mixture of *F. verticillioides* toxins.

Compared to terrestrial animals, the toxicological effects of mycotoxins are barely studied in fish (Gonçalves et al. 2020c). The majority of the few fish studies that have been published have often focussed on one single mycotoxin (Anater et al. 2016). Due to its sensitivity, several studies on the toxicological impact of DON have been performed in rainbow trout (*Oncorhynchus mykiss*) (Koletsi et al. 2021; Hooft and Bureau 2021). With the exception of one study on FB_1_ (Carlson et al. 2001), no studies on *F. verticillioides* toxins have been completed in trout. FB_1_ altered the metabolism of sphingolipids in rainbow trout (Carlson et al. 2001), but no information was presented regarding its effect on growth performance measurements. In other farmed fish species, fumonisins impaired growth (seabream, (Gonçalves et al. 2020a); turbot, (Gonçalves et al. 2020b); African catfish, (Gbore et al. 2010); Nile tilapia, (Tuan et al. 2003); channel catfish, (Lumlertdacha et al. 1995; Yildirim et al. 2000). Despite its frequent occurrence in European aquafeeds (Koletsi et al. 2021), information on fusaric acid toxicity in farmed fish species is lacking. Finally, information on the interactions between different types of toxins (co-exposure) in fish is minimal. In zebrafish, it was observed that co-exposure to different combinations of toxins also bring about different toxicological effects. The toxicological effects of FB_1_ and aflatoxin B_1_ (AFB_1_) were additive (no interaction) (Di Paola et al. 2022). Similarly, zearalenone (ZEN) and FB_1_ effects were additives (Yang et al. 2021), whereas the effects of AFB_1_ and DON were synergistic, and the effects of DON, ZEN and AFB_1_ were antagonistic (Zhou et al. 2017). To our knowledge, only two in vivo feeding experiments were reported on farmed fish species, where synergistic toxicological effects of FB_1_ and moniliformin were found in catfish (Yildirim et al. 2000) and AFB_1_ and ZEN in rainbow trout (Ghafarifarsani et al. 2021).

Therefore, this experiment aimed to determine whether the toxicological effects of deoxynivalenol (DON) produced by *F. graminearum* are altered by the co-exposure to a mixture of toxins produced by *F. verticillioides* (FU_mix_; fusaric acid and FB_1_, FB_2_ and FB_3_) in rainbow trout. This was assessed by measuring growth performance and histopathological measurements in the liver and gastrointestinal tract under restrictive and *ad libitum* exposure.

## Materials and methods

The current study (project number: AVD2330020198084) was approved by the Central Committee on Animal Experiments (CCD) of The Netherlands. All experimental procedures were carried out following the Dutch law on the use of animals for scientific purposes. The feeding trial was performed at the experimental facilities of the Alltech Coppens Aqua Centre (Leende, The Netherlands).

### Experimental design and diets

In the experiment, four diets were studied according to a 2 × 2 factorial design. The first factor was the contamination level of DON produced by *Fusarium graminearum*. The intended contrast in DON exposure levels was 0 and 2000 µg/kg feed on a fresh basis (CON versus DON diets). The second factor was the contamination level of the toxin mixture (FU_mix_; fusaric acid and FB_1_, FB_2_ and FB_3_) produced by *Fusarium verticillioides*. The intended contrast in FU_mix_ exposure was aimed to have an FB_1_ content of 0 versus 8000 µg/kg feed on a fresh basis (CON versus FU_mix_ diets). These contrasts in contamination levels were created by exchanging toxin-free ingredients with artificially contaminated ingredients (rice and cracked corn for the DON and FU_mix_ exposure, respectively). Consequently, the four experimental diets: CON-CON (DON = 0 µg/kg, FU_mix_ = 0 µg/kg), CON-FU_mix_ (DON = 0 µg/kg, FU_mix_ = 12,000 µg/k), DON-CON (DON = 2800 µg/kg, FU_mix_ = 180 µg/kg) and DON-FU_mix_ (DON = 2500 µg/kg, FU_mix_ = 13,500 µg/kg) were nutritionally identical (isoenergetic and isonitrogenous) and only differed in the mycotoxin profile (Table 1). Diets were produced by Research Diet Services (Wijk bij Duurstede, The Netherlands) as 2 mm extruded pellets.

**Table 1:** Ingredient composition, proximate, and mycotoxin analysis of the experimental diets: without DON or other mycotoxins (CON-CON), without DON but contaminated with a mixture of toxins produced by *Fusarium verticillioides*: fusaric acid and FB_1_, FB_2_ and FB_3_ (CON-FU_mix_), contaminated with DON alone produced by *Fusarium graminearum* (DON-CON), and co-contaminated with all toxins produced by *F. graminearum* and *F. verticillioides* (DON-FU_mix_)

Ingredients inclusion (%)	Experimental diets			
	CON-CON	CON-FU_mix_	DON-CON	DON-FU_mix_
Wheat	38.42	38.42	38.42	38.42
Soybean meal	25.00	25.00	25.00	25.00
LT fishmeal	12.93	12.93	12.93	12.93
Fish oil	11.98	11.98	11.98	11.98
Blood meal	7.94	7.94	7.94	7.94
Clean cracked corn	1.60	–	1.60	–
Contaminated cracked corn	–	1.60	–	1.60
Clean rice	0.26	0.26	–	–
Contaminated rice	–	–	0.26	0.26
Monocalcium phosphate	0.66	0.66	0.66	0.66
DL-methionine liquid	0.16	0.16	0.16	0.16
Choline chloride liquid	0.18	0.18	0.18	0.18
Premixes^a^	0.88	0.88	0.88	0.88
Analysed nutrient composition (%)^b^				
Dry matter	94.0	94.4	94.5	94.4
Protein	37.6	37.5	37.6	37.7
Fat	15.8	16.0	15.8	15.9
Ash	6.3	6.2	6.1	6.3
Gross energy (MJ/kg)	22.6	22.6	22.3	22.4
Mycotoxin concentration (µg/kg)^b,c^				
Deoxynivalenol (DON)	–	–	2809	2495
Fusaric acid	–	2696	183	3281
Fumonisin B_1_ (FB_1_)	–	7599	–	8557
Fumonisin B_2_ (FB_2_)	–	1199	–	1163
Fumonisin B_3_ (FB_3_)	–	485	–	526

^a^Commercial premix from Alltech Coppens to meet (NRC 2011) requirements of rainbow trout

^b^On dry matter basis, CON-CON diet contained only Enniatin A/A1 0.95 µg/kg

^c^In the main text, the rounded levels are mentioned e.g. DON 2800 and 2500 µg/kg. Rounded FU_mix_ totals are, respectively, 12,000, 180 and 13,500 µg/kg

The artificially contaminated ingredients were produced by fermentation with mycotoxin-producing fungi at the Laboratory of Mycotoxins and Mycology, Department of Biological Sciences, College of Agriculture Luiz de Queiroz, University of São Paulo. Rice inoculated with a *F. graminearum* isolate was fermented to produce DON-contaminated rice and cracked corn with a *F. verticillioides* isolate to produce the FU_mix_. Briefly, the Erlenmeyer flasks of 500 mL volume were used each containing 100 g of rice or corn. At least 2 h before the sterilization, 40 mL of distilled water was added to the flask and mixed with rice or corn. The sterilization was performed at 121 °C for 1 h (CS -75, Prismalab, Rio de Janeiro, RJ, Brazil). Thereafter, the flasks were left to cool down before inoculation. The sterilized ingredients were inoculated with 2 mL of conidia suspension with 10^6^ conidium/mL of either *Fusarium graminearum* or *Fusarium verticillioides.* The incubation was carried out for 25 days at a constant temperature of 25 °C in static conditions for the DON and FU_mix_ production. After incubation, the fermented ingredients containing the respective mycotoxins were oven dried at 50 °C. After drying, the ingredients were ground in a mill with a 0.85-mm sieve. For the control treatments, non-inoculated rice and/or cracked corn of the same batches were used.

The mycotoxin content of the spiked ingredients and experimental diets were analysed with liquid chromatography/tandem mass spectrometry (LC–MS/MS) at the Alltech 37 + mycotoxin laboratory (Dunboyne, Ireland; ISO/IEC 17025:2005 accredited). The analysed DON content in rice was 768 mg/kg on as is basis and the FB_1_ content in corn 220 mg/kg on as is basis. Based on these analysed contents and the targeted contrasts in DON (2000 µg/kg) and FB_1_ (8000 µg/kg) between diets, the inclusion levels of clean and contaminated rice and cracked corn were set at, respectively, 0.26 and 1.60% in the diets (Table 1). In the experimental diets, the targeted levels of DON and FB_1_ (in the FU_mix_) were reached; however, the DON-CON diet contained some traces of fusaric acid (Table 1).

### Husbandry

Rainbow trout (*Oncorhynchus mykiss*) with an average initial body weight of approximately 7 g were maintained in a recirculating aquaculture system (RAS) for 8 weeks. The housing conditions were similar to those of a previous in vivo experiment (Koletsi et al. 2022). Fish were purchased from a commercial trout farm (Mohnen Aquaculture GmbH, Germany) 1 week prior to the start of the experiment during which they were fed a standard commercial trout diet. Ten tanks were each stocked with 30 fish. Tanks were randomly assigned to one of the experimental diets. The CON-CON and CON-FU_mix_ diets were tested in duplicate and the DON-CON and DON-FU_mix_ diets in triplicate. Fish were housed at a temperature of 14 ± 0.5 °C. The applied photoperiod was 17 h of light and 7 h of darkness. Water quality was monitored and maintained within the optimal range for trout. In the outlet water of the tanks, the measured pH ranged from 7.0 to 8.5, NH_4_^+^ was below 1 mg/L, NO_2_^−^ was below 0.5 mg/L, and oxygen (O_2_) was above 8 mg/L. During the whole experiment, fish were hand-fed twice per day. During the first 6 weeks of the experiment, trout were fed restrictively in order to measure the direct impact of toxins. In this period, the feeding level was based on the metabolic body weight of the fish (12 g/kg^0.8^/d). During the last 2 weeks of the experiment, fish were fed *ad libitum* for 1 h during each meal to determine the potential impact of the tested mycotoxins on feed intake capacity. When uneaten pellets remained on the bottom of the tank or floating on the water’s surface for more than 10 min or when the feeding time of one hour was over, the feeding was stopped, and it was assumed that the fish had reached satiation. During both feeding periods, uneaten pellets were removed by siphoning after feeding was stopped and counted to accurately determine feed consumption.

### Sampling

The sampling scheme and the processing of samples were similar to those applied in our previous in vivo experiment (Koletsi et al. 2022). Briefly, tank biomass measurements were performed at the start of the experiment, the end of the restrictive feeding period (week 6) and the end of the *ad libitum* feeding period (week 8) to calculate growth performance indicators. At the start of the experiment, 20 fish from the initial population were removed, and at the end of the restrictive exposure (week 6), five fish per tank were euthanised and stored at − 20 °C. These samples were used for body composition measurements to calculate protein and energy retention. Additionally, for histopathological analysis, tissue samples from the liver (two sections per fish) and one section of each gastrointestinal tract segment (pyloric caeca, midgut, and hindgut) were collected from six fish of the initial population and from two fish per tank at week 1 and week 6 of restrictive feeding period and at the end of the *ad libitum* feeding period (week 8). These tissue samples were placed into embedding cassettes, fixed by immersion in 10% neutral buffered formaldehyde for three days at room temperature and afterwards transferred to 70% ethanol until further processing. Before collecting these tissue samples, body weight, liver weight and body length were recorded in these fish.

### Chemical analysis

Fish carcass and feed samples were analysed for dry matter, crude protein and fat, ash content and gross energy by Nutricontrol (Veghel, The Netherlands) as described previously (Koletsi et al. 2022).

### Histological analysis

Liver and intestinal tissue samples were dehydrated in a tissue processor and embedded in paraffin wax according to standard histological procedures. Tissue blocks were cut into 5 μm thick paraffin sections, mounted onto microscope slides and stored until further processing. Thereafter, liver sections were stained with two separate techniques: Haematoxylin and Eosin (H&E) to colour the cell nuclei and structure, and periodic acid-Schiff’s (PAS) reagent to distinguish glycogen from lipid vacuolisation. The gastrointestinal tract sections were stained with Alcian blue (pH 2.5) followed by Crossman. All stained slides were pictured with a Leica DM6 microscope (Leica Microsystems, Wetzlar, Germany). Liver pictures (*n* = 10 per fish) were further evaluated using the semi-quantitative scoring system described by (Koletsi et al. 2022). The gastrointestinal tract pictures were imported in ImageJ software (version 1.53q) (Schindelin et al. 2012). With the ROI manager function of ImageJ, on 10 well-oriented (simple) mucosal fold units per fish (*n* = 10 per fish) the following indicators were measured as previously described (Koletsi et al. 2022): mucosal fold width, mucosal fold height, lamina propria width, enterocyte width, supranuclear vacuoles width and goblet cell density.

### Calculations and statistics

The following measurements, growth (g/d), specific growth rate (SGR, %/d) and performance; feed conversion ratio (FCR), hepatosomatic index (HSI, %), condition factor (K), retained protein (g/fish), protein retention efficiency (%), retained energy (MJ/fish), energy retention efficiency (%), were calculated separately for each feeding period (6 weeks restrictive and 2 weeks *ad libitum* feeding) according to previously established equations (Koletsi et al. 2022).

A two-way ANOVA was used to analyse the growth performance measurements for the effect of DON supplementation, FU_mix_ supplementation and their interaction effect (FU_mix_ and DON). Before ANOVA, Levene’s test was used to determine whether the variance of the data was homogeneous. The Kolmogorov–Smirnov test was applied to determine whether the distribution of residuals was normal. For non-normally distributed data, a non-parametric test, Kruskal–Wallis, was applied to test the FU_mix_ effect and the DON effect, although this model could not test the interaction effect. Histological data (*n* = 600 per time point) from each segment of the gastrointestinal tract (pyloric caeca, midgut and hindgut) and ordinal measurements in the liver: glycogen and lipid (scores of 1, 2 and 3) and necrosis (scores of 0, 1, 2 and 3) were analysed with a mixed-effect model, multinomial logistic regression using the fish as the random effect. The fixed variables tested were the effects of FU_mix_, DON, time (week 1, 6 and 8) and their interactions. Liver binomial data (nuclei pyknosis and pleomorphism, necrosis, haemorrhage, inflammation) were expressed as percentages (%) and analysed with a mixed binary logistic regression model including FU_mix_, DON, time and their interactions as fixed effects and the fish as a random effect. Statistical significance was tested at a probability level below 0.05 (*p* ≤ 0.05), while *p*-values between 0.1 and 0.05 (0.1 > *p* ≥ 0.05) were defined as close to statistical significance and reported as tendencies. All data were statistically analysed in the IBM Statistical Package for the Social Sciences (SPSS) program (v 23.0; New York, NY, USA).

## Results

### Growth performance

During the 8-week experiment, no mortality, abnormal behaviour or issues with feed acceptance were noted.

### Restrictive feeding period

During the restrictive feeding period, growth and FCR were affected by both FU_mix_ exposure (*p* ≤ 0.01) and DON exposure (*p* ≤ 0.001) (Table 2). Trout fed the FU_mix_ diets had lower growth than those fed the diets without the FU_mix_. Growth of trout fed the DON diets was lower than that of trout fed the diets without DON. The decline in growth due to the presence of DON (0.05 g/d) was identical at the diet level with and without the FU_mix_ (Table 2), indicating that the effects of DON and FU_mix_ were additive (no interaction). FCR was increased when diets were contaminated with the FU_mix_ and the increase was even higher when contaminated with DON compared to their controls. During the restrictive feeding period, the HSI was only affected by FU_mix_ (*p* ≤ 0.01), being lower in trout fed the diets with the FU_mix_ compared to trout fed the diets without the FU_mix_. The condition factor was only influenced by DON (*p* ≤ 0.05) and was lower in fish fed diets with DON compared to those fed the diets without DON. Finally, DON was also the only factor that affected protein retention (*p* ≤ 0.001), protein retention efficiency (*p* ≤ 0.001), energy retention (*p* ≤ 0.05) and energy retention efficiency (*p* ≤ 0.05) (Table 2). Trout fed the DON-contaminated diets retained less protein and less energy compared to trout fed diets without DON. FU_mix_ had no impact on metrics of retained protein and energy (Table 2).

**Table 2:** The effects of FU_mix_ exposure, DON exposure and their interaction on growth performance measurements of rainbow trout during a 6-week restrictive feeding period

**Measurements**^**b**^	**Experimental diets**^**a**^					*p*-value		
	CON-CON	CON-FU_mix_	DON-CON	DON-FU_mix_	SEM	FU_mix_	DON	FU_mix_ × DON
Initial BW (g)	7.1	7.5	7.3	7.3	0.16	NS	NS	NS
Final BW (g)	26.4	25.5	24.6	23.4	0.22	**	***	NS
Growth (g/d)	0.48	0.45	0.43	0.40	0.01	**	***	NS
FCR	0.79	0.85	0.89	0.95	0.01	**	***	NS
HSI (%)^c^	2.6	1.6	2.3	1.4	0.43	**	NS	–
Condition factor (K)	1.30	1.28	1.22	1.18	0.03	NS	*	NS
Retained protein (g/fish)	3.1	2.9	2.5	2.4	0.07	NS	***	NS
Protein retention efficiency (%)	52.9	50.6	43.6	41.3	1.33	NS	***	NS
Retained energy (MJ/fish)	0.16	0.16	0.15	0.15	0.004	NS	*	NS
Energy retention efficiency (%)	47.2	45.3	43.7	42.7	1.18	NS	*	NS

The measured levels of DON and FU_mix_ (fusaric acid and FB_1_, FB_2_ and FB_3_) in the diets are given in Table 1

Values presented are means based on *n* = 2 for the diets CON-CON and CON-FU_mix_ and *n* = 3 for the diets DON-CON and DON-FU_mix_

FU_mix_, a mixture of toxins produced by *Fusarium verticillioides*: fusaric acid and FB_1_, FB_2_ and FB_3_

DON, a toxin produced by *Fusarium graminearum*

*p ≤ 0.05; **p ≤ 0.01; ***p ≤ 0.001

^a^CON-CON, diet without DON and without FU_mix_ contamination; CON-FU_mix_, diet without DON and with FU_mix_ contamination; DON-CON, diet with DON and without FU_mix_ contamination; DON-FU_mix_, diet with DON and with FU_mix_ contamination

^b^*BW* body weight, *FCR* feed conversion ratio on dry matter basis, *HSI* hepatosomatic index, *SEM* standard error of means, *NS* not significant

^c^Analysed with a non-parametric test (Kruskal–Wallis) for FU_mix_ and DON effect, where “–” FU_mix_ × DON was not applicable

### *Ad libitum* feeding period

During the *ad libitum* feeding period, the feed intake, growth and FCR of rainbow trout were only influenced by the DON treatment (*p* ≤ 0.01; Table 3), not by FU_mix_ treatment and the interaction. Trout fed the DON-contaminated diets had lower feed intake, lower growth and higher FCR compared to those fed the DON-free diets (Table 3). At the end of the *ad libitum* feeding period, both DON and FU_mix_ treatments did not affect the condition factor. Liver weight (HSI) was reduced in fish fed diets containing the FU_mix_ compared to those fed diets without the FU_mix_ (*p* ≤ 0.01; Table 3).

**Table 3:** The effects of FU_mix_ exposure, DON exposure and their interaction on growth performance measurements of rainbow trout during a 2-week *ad libitum* feeding period

**Measurements**^**b**^	**Experimental diets**^**a**^					p-value		
	CON-CON	CON-FU_mix_	DON-CON	DON-FU_mix_	SEM	FU_mix_	DON	FU_mix_ × DON
Final BW (g)	52.1	51.2	41.2	39.9	1.12	NS	***	NS
Growth (g/d)	1.71	1.70	1.11	1.10	0.07	NS	***	NS
Feed intake (g/fish/d)	1.57	1.60	1.22	1.25	0.04	NS	***	NS
FCR	0.86	0.89	1.04	1.08	0.04	NS	**	NS
HSI (%)	2.1	1.4	2.2	1.9	0.18	**	NS	NS
Condition factor (K)	1.3	1.3	1.3	1.2	0.04	NS	NS	NS

The measured levels of DON and FU_mix_ (fusaric acid and FB_1_, FB_2_ and FB_3_) in the diets are given in Table 1

Values presented are means based on *n* = 2 for the diets CON-CON and CON- FU_mix_ and *n* = 3 for the diets DON-CON and DON-FU_mix_

FU_mix_, a mixture of toxins produced by *Fusarium verticillioides*: fusaric acid and FB_1_, FB_2_ and FB_3_

DON, a toxin produced by *Fusarium graminearum*

*p ≤ 0.05; **p ≤ 0.01; ***p ≤ 0.001

^a^CON-CON, diet without DON and without FU_mix_ contamination; CON-FU_mix_, diet without DON and with FU_mix_ contamination; DON-CON, diet with DON and without FU_mix_ contamination; DON-FU_mix_, diet with DON and with FU_mix_ contamination

^b^*BW* body weight, *FCR* feed conversion ratio on dry matter basis, *HSI* hepatosomatic index, *SEM* standard error of means, *NS* not significant

## Histopathological assessment of liver and gastrointestinal tract

### Liver

**Fig. 1:**
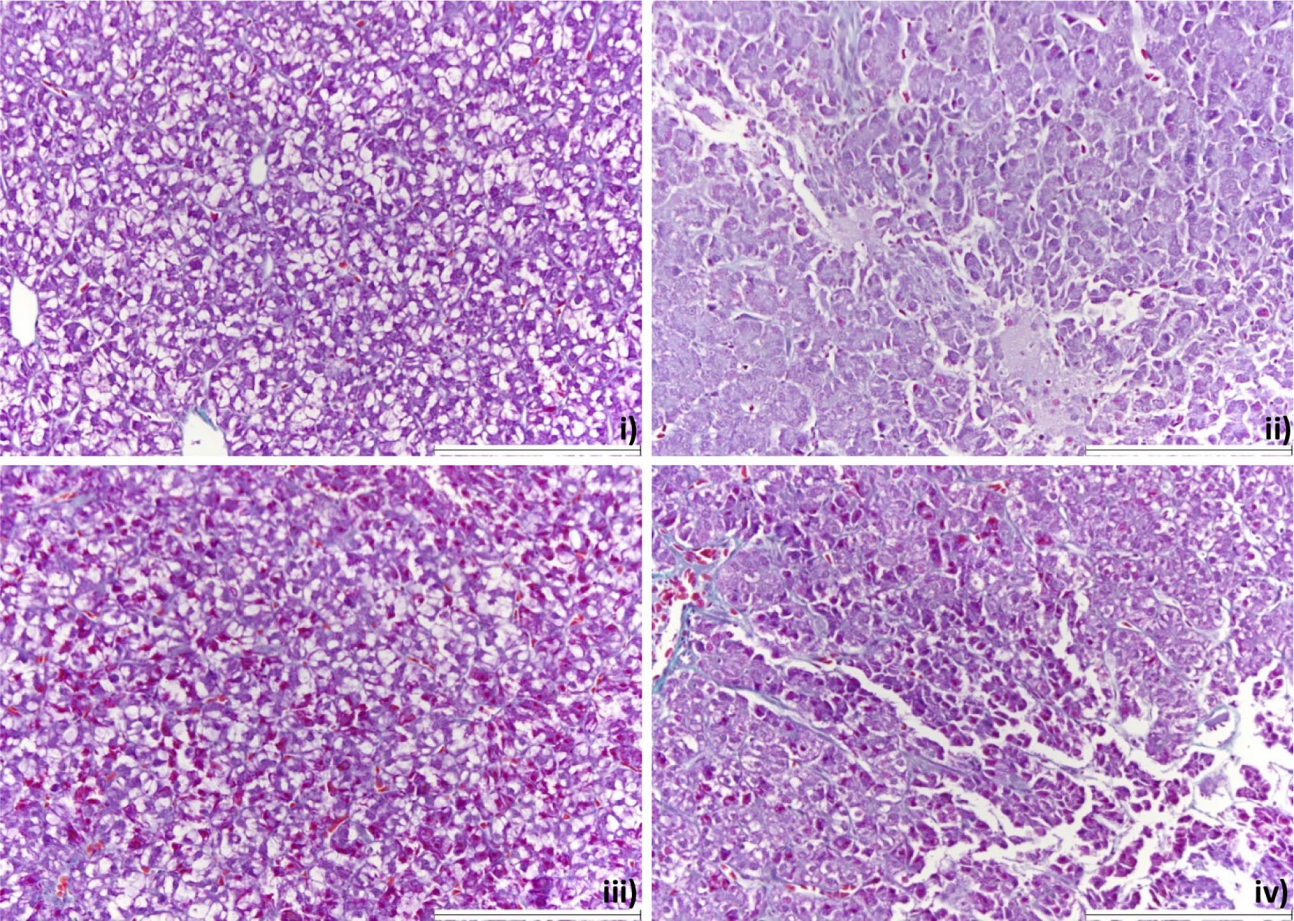
Examples of histological sections of the liver at the end of the experiment (week 8) from rainbow trout fed: (i) CON-CON diet (DON = 0, FU_mix_ = 0), (ii) CON-FU_mix_ diet (DON = 0, FU_mix_ = 12,000), (iii) DON-CON diet (DON = 2800, FU_mix_ = 180) and (iv) DON-FU_mix_ diet (DON = 2500, FU_mix_ = 13,500). Staining: PAS-crossman; magnification: × 20; white scale bar = 200 µm

The qualitative assessment of the liver histology did not show severe liver damage, but only some minor changes. Some examples of minor changes are given in Fig. 1. where panel (i) shows an unaffected liver; panel (ii) a liver with necrotic areas; panel (iii) a liver with scattered blood cells; and panel (iv) a liver with both necrotic areas and scattered blood cells.

The semi-quantitative assessment (Table 4) showed that for pyknotic nuclei, all scores were 0 in week 8 only for the DON-CON diet. For inflammation, all scores during the restrictive feeding period (weeks 1 and 6) were 0 for all diets. After *ad libitum* feeding (week 8), however, 23 and 27% inflammation spots were found for the FU_mix_ contaminated diets, compared to 7% in the DON-CON diets and 0% in the CON-CON diet. Due to the presence of 0 scores in one or multiple combinations of diets and weeks, the effects of DON and FU_mix_ could not be estimated for pyknotic nuclei and inflammation (Table 4).

**Table 4:** Histological assessment in trout livers with the effects of DON, FU_mix_, time and their interactions after feeding the experimental diets restrictively for 6 days (week 1) and 40 days (week 6) and *ad libitum* for 15 days (week 8)

**Week**		**Experimental diets**					***p*-value**					
		**CON-CON**	**CON-FU**_**mix**_	**DON-CON**	**DON-FU**_**mix**_	**FU**_**mix**_	**DON**	**time**	**FU**_mix_*DON	**FU**_mix_*time	DON*time	**FU**_mix_*DON*time
**Vacuolization score**												
Glycogen^a^	1	2.0	1.8	1.7	1.6							
	6	1.8	1.5	1.7	1.9	*	NS	***	NS	***	NS	NS
	8	1.9	2.7	2.0	2.5							
Lipid^a^	1	2.0	2.0	1.9	2.0							
	6	2.2	2.0	2.1	1.8	NS	NS	NS	NS	NS	NS	NS
	8	1.7	1.3	1.9	1.8							
**Nuclei characteristics**												
Pyknotic (%)	1	15	23	5	10							
	6	18	20	20	3	–^b^	–	–	–	–	–	–
	8	25	25	0	20							
Pleomorphic (%)	1	3	43	13	25							
	6	48	28	47	3	**	NS	***	NS	***	NS	**
	8	72	3	62	63							
**Pathological indicators**												
Necrosis (%)	1	13	8	23	15							
	6	18	20	30	45	NS	**	****	NS	NS	NS	NS
	8	25	15	20	27							
Necrosis score^a^	1	0.2	0.1	0.3	0.2							
	6	0.3	0.3	0.5	0.9	NS	**	*	NS	NS	NS	NS
	8	0.3	0.2	0.3	0.5							
Haemorrhage (%)	1	30	15	52	45							
	6	18	35	32	23	NS	NS	NS	NS	NS	NS	NS
	8	15	13	13	23							
Inflammation (%)	1	0	0	0	0							
	6	0	0	0	0	–^b^	–	–	–	–	–	–
	8	0	23	7	27							

Total number of observations was *n* = 600, apart from glycogen and lipid vacuolisation *n* = 585

*NS* not significant

*p ≤ 0.05; **p ≤ 0.01; ***p ≤ 0.001; ****p ≤ 0.1

^a^Glycogen, lipid vacuolisation and necrosis scores were analysed with a generalised linear mixed model by using multinomial logistic regression and the other pathological indicators by using binary logistic regression in which frequencies were used. In the table, however, descriptive means are shown

^b^For pyknotic nuclei and inflammation, it was not possible to estimate model coefficients, since one or more diet-week combinations show 0% affected spots

During the restrictive feeding period (weeks 1 and 6), the glycogen vacuolization score was similar for all diets. At the end of the* ad libitum* feeding period (week 8), however, the glycogen vacuolization score increased only in the trout fed the diets containing the FU_mix_ (interaction *p* ≤ 0.001). The effect of DON on glycogen vacuolization was not present (Table 4). Lipid vacuolization did not change over time and was unaffected by both dietary treatments. Regarding the percentage of pleomorphic nuclei, the 3-way interaction effect was present (*p* ≤ 0.01), but there were no clear patterns of the effects of DON and FU_mix_ over time (Table 4). In livers of DON-fed trout, the risk on the higher order necrosis scores was increased compared to livers of trout not exposed to DON. Necrosis was also present in trout fed the CON-CON and CON-FU_mix_ diet, although with a low average score (ranging from 0.1 to 0.3) and a lower percentage of liver parts affect. Time also affected the liver necrosis score (*p* ≤ 0.01), being the highest at week 6 (Table 4). The percentage of haemorrhage was not significantly affected by the dietary treatments and time (*p* > 0.05; Table 4).

### Gastrointestinal tract

**Fig. 2:**
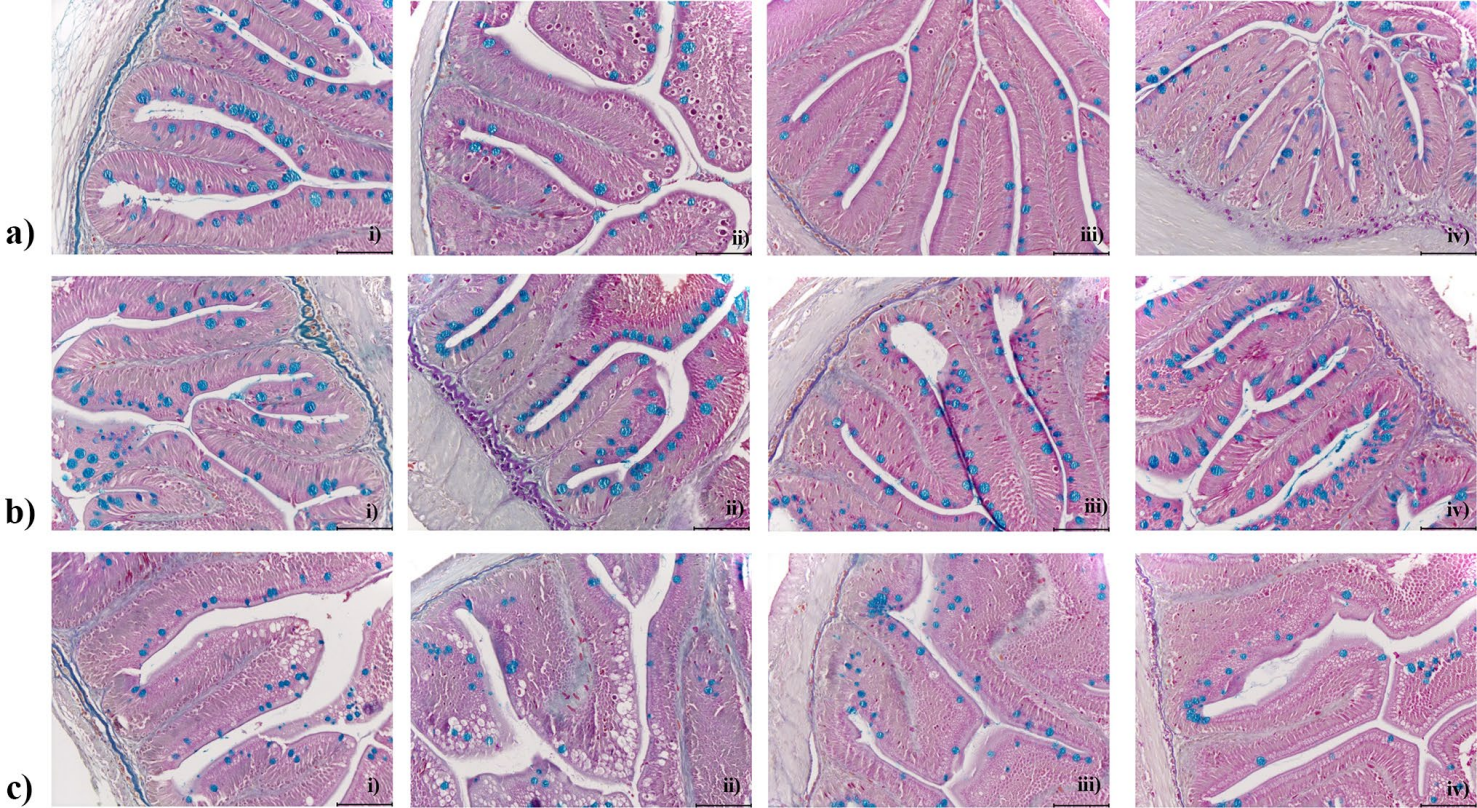
Representative examples of histological sections of the intestinal folds at the end of the experiment (week 8) in a pyloric caeca, b midgut, and c hindgut of rainbow trout fed: (i) CON-CON diet (DON = 0, FU_mix_ = 0), (ii) CON-FU_mix_ diet (DON = 0, FU_mix_ = 12,000), (iii) DON-CON diet (DON = 2800, FU_mix_ = 180), and (iv) DON-FU_mix_ diet (DON = 2500, FU_mix_ = 13,500). Staining: Alcian blue-crossman; magnification: × 20; black scale bar = 100 µm

The statistical outcome of the semi-quantitative histological assessment in the gastrointestinal tract of rainbow trout response to FU_mix_, DON, time and their interactions (3-way and 2-way) is presented in Table 5, showing mild histopathological changes indicated by a few 2-way significant interactions. Figure 2 displays examples of the intestinal folds from the pyloric caeca, midgut and hindgut, collected at the end of the experiment (week 8). Similarly in the qualitative analyses (Fig. 2), no notable histological alterations were observed in the gastrointestinal tract.

**Table 5:** Effects of DON, FU_mix_, time and their interactions on histological measurements in pyloric caeca, midgut and hindgut of rainbow trout after feeding the experimental diets restrictively for 6 days (week 1) and 40 days (week 6) and *ad libitum* for 15 days (week 8)

		**Experimental diets**				***p*-value **							
Week	Week	CON-CON	CON-FU_mix_	DON-CON	DON-FU_mix_	SEM1	FU_mix_	DON	time	FU_mix_ × DON	FU_mix_ × time	DON × time	FU_mix_ × DON × time
**Mucosal fold width (µm)**													
Pyloric	1	129	127	138	128								
	6	126	141	124	130	8.7	NS	NS	**	NS	NS	NS	NS
	8	169	145	136	149								
Midgut	1	122	131	132	134								
	6	134	150	128	131	6.2	NS	NS	****	NS	**	****	NS
	8	145	130	150	131								
Hindgut	1	130	139	118	129								
	6	184	132	132	120	14.5	****	****	NS	NS	****	NS	NS
	8	156	122	144	133								
**Mucosal fold height (µm)**													
Pyloric	1	301	317	311	304								
	6	403	399	378	372	28.7	NS	NS	***	NS	NS	NS	NS
	8	490	425	414	452								
Midgut	1	306	379	350	329								
	6	343	371	377	314	28.1	NS	NS	***	****	NS	NS	NS
	8	458	425	410	374								
Hindgut	1	380	433	397	404								
	6	459	390	323	303	52.3	NS	NS	NS	NS	NS	****	NS
	8	432	394	474	486								
**Lamina propria width (µm)**													
Pyloric	1	12	12	14	12								
	6	16	17	14	14	2	NS	NS	NS	NS	NS	NS	NS
	8	18	12	13	15								
Midgut	1	15	14	15	15								
	6	18	19	19	18	1.6	NS	NS	**	NS	NS	NS	NS
	8	14	15	17	16								
Hindgut	1	12	13	10	11								
	6	16	16	14	14	1.6	NS	NS	*	NS	NS	NS	NS
	8	13	12	14	13								
**Enterocyte width (µm)**													
Pyloric	1	66	64	72	66								
	6	65	76	67	74	4.7	NS	NS	***	NS	****	NS	NS
	8	87	80	77	79								
Midgut	1	56	65	66	68								
	6	72	82	60	69	5	NS	NS	***	NS	***	*	NS
	8	84	69	87	71								
Hindgut	1	51	65	52	71								
	6	80	69	61	67	7.5	NS	NS	****	NS	**	NS	NS
	8	80	60	76	66								
**Supranuclear vacuole width (µm)**													
Pyloric	1	50	51	51	49								
	6	44	46	42	41	4.5	NS	NS	*	NS	NS	NS	NS
	8	58	52	44	52								
Midgut	1	49	52	44	50								
	6	44	48	42	43	3.7	NS	NS	NS	NS	NS	NS	NS
	8	47	47	46	44								
Hindgut	1	67	59	54	46								
	6	86	45	57	40	8.1	**	*	NS	NS	NS	NS	NS
	8	63	50	54	54								
**Goblet cell density (per µm fold height)**													
Pyloric	1	0.05	0.04	0.03	0.02								
	6	0.07	0.05	0.04	0.04	0.012	*	*	****	**	NS	NS	NS
	8	0.08	0.02	0.03	0.04								
Midgut	1	0.08	0.07	0.07	0.07								
	6	0.09	0.11	0.12	0.12	0.01	NS	****	***	NS	NS	NS	NS
	8	0.07	0.07	0.09	0.08								
Hindgut	1	0.04	0.06	0.05	0.05								
	6	0.05	0.04	0.04	0.04	0.009	NS	NS	NS	NS	NS	NS	NS
	8	0.04	0.04	0.04	0.05								

*NS* not significant

*p ≤ 0.05; **p ≤ 0.01; ***p ≤ 0.001; ****p ≤ 0.1

^a^Pooled standard error of means: SEM (pyloric: *n* = 544, midgut: *n* = 481, hindgut: *n* = 285)

The semi-quantitative assessment of the intestinal histology showed that none of the indicators was affected by the 3-way interaction effect between DON, FU_mix_ and time (Table 5). The enterocyte width in the midgut was the only intestinal indicator that was affected by the interaction between DON and time (*p* ≤ 0.05), which was related to an alteration in the effect of DON between week 1 and week 6 of the restrictive feeding period (Table 5). Mucosal fold width in the midgut and enterocyte width in the midgut and hindgut were affected by the 2-way interaction between time and FU_mix_ (*p* ≤ 0.01). These indicators were higher during week 1 and week 6 in fish fed diets containing FU_mix_, while during week 8 (the end of the *ad libitum* feeding period), these width measurements were reduced in fish fed diets containing FU_mix_ (Table 5). The goblet cell density of the pyloric caeca was the only indicator with an interaction effect between DON and FU_mix_ (*p* ≤ 0.01). Fish fed diets containing toxins had a similar goblet cell density in the caeca, but these densities were lower compared to the fish fed the toxin-free diet (CON-CON; Table 5). No interaction effect between DON and FU_mix_ was noted in any of the other intestinal indicators measured. The supranuclear vacuole width in the hindgut was affected by both DON (*p* ≤ 0.05) and FU_mix_ (*p* ≤ 0.01), without an interaction. Trout exposed to DON and FU_mix_ had a reduced supranuclear vacuole width (Table 5)*.*

## Discussion

The current study investigated, via a 2 × 2 factorial design, the impact of individual and combined effects of *Fusarium graminearum*- and *Fusarium verticillioides*-produced toxins on growth performance and histology of the gastrointestinal tract of rainbow trout. The first factor was DON contamination produced by *F. graminearum* (DON), and the second factor was the mixture of the toxins: fusaric acid and FB_1_, FB_2_ and FB_3_ (FU_mix_) produced by *F. verticillioides.* Therefore, the four experimental diets had a contrast without and with DON contamination (CON versus DON) and without and with FU_mix_ contamination (CON versus FU_mix_).

The restrictive feeding period revealed a direct impact of DON at a dose of 2700 µg/kg on growth, FCR, protein and energy retention. At half DON dose, an earlier study (Koletsi et al. 2022) measured the direct impacts of DON on protein and energy retention. These observations were not influenced by a reduction in feed intake since the restrictive feeding regime aimed to offer the same amount of feed in all treatments. Therefore, any change in the growth performance indicators was associated with the mode of action of DON (e.g., inhibition of protein synthesis). During the *ad libitum* feeding period, the accurate monitoring of feed consumption by subtracting the uneaten pellets showed that DON exposure reduced feed intake in trout. This is in contrast to a previous in vivo study in trout with the same experimental design (Koletsi et al. 2022), where no effect of DON on feed intake was present. This difference might be explained by exposure to DON at a dose of 2700 µg/kg, which was higher than that in the previous study (Koletsi et al. 2022). The current DON dose resulted in an estimated daily intake (EDI) of 0.104 μg DON/g BW/day during the *ad libitum* feeding period, whereas in our earlier study, the EDI of DON was only 0.044 μg/g BW/day (Koletsi et al. 2022). Most likely, the differences in DON exposure level may explain the differences between studies regarding appetite. The current observation of a reduced feed intake is in line with studies in trout applying an *ad libitum* feeding period of 8 weeks (Hooft et al. 2011, 2019a, b; Ryerse et al. 2016; Hooft and Bureau 2017; Gonçalves et al. 2018b). The combination of the dose of DON and experimental duration should, therefore, be considered when investigating statistical differences in feed intake.

Considering the higher dose of DON (2700 µg/kg) applied in the current study, it was expected that alterations in the liver histological measurements would be more severe compared to the ones reported in an earlier study (Koletsi et al. 2022) even at half DON dose. Other DON studies did not detect histopathological changes in the liver of trout (Matejova et al. 2014) (Hooft et al. 2019a) and of red tilapia (Tola et al. 2015).In contrast, histopathological changes were observed qualitatively in trout (Hooft et al. 2011; Gonçalves et al. 2019) and quantitatively in carp (Pietsch et al. 2014; Pietsch and Burkhardt-Holm 2015). The minor changes in the current study and variability between studies in DON impact on the liver might be linked to factors such as differences in the power of the study (in the current study, 4 or 6 fish were sampled for histology per treatment at each time point); variability inside the tank between fish in EDI of DON due to differences in feed intake; the occurrence of unknown co-exposure with other mycotoxins (over the years, the detection methods of mycotoxins have evolved; new toxins are discovered and analysed); differences in experimental conditions and genetic background and life history of the experimental fish. The minor/mild histological changes induced by DON on gut histology are in line with an earlier study (Koletsi et al. 2022). The absence/minor effect of DON on trout intestinal tissues may be related to the rapid absorption of the toxin in the upper part of the gastrointestinal tract and distribution to the liver within 1 h (Bernhoft et al. 2017). While the gastrointestinal tract was consistently unaffected by DON in our studies, other studies (Koletsi et al. 2022) reported alterations of histological measurements in the liver, where DON is eliminated at the half-life within 6.2 h (Bernhoft et al. 2017).

Regarding the second factor in this study, FU_mix_, it is not possible to estimate the contribution of each separate toxin present in the mixture produced by *F. verticillioides* (fusaric acid and FB_1_, FB_2_ and FB_3_) to the total effect of the mixture. Information on fusaric acid and FB_3_ effects on fish is absent. In the EU recommendation for toxins, FB_1_ and FB_2_ are summed with a current limit of 10,000 µg/kg (Commission 2006). In the FU_mix_ contaminated diets in the current study, the mean FB_1_ and FB_2_ level was ~ 9000 µg/kg feed, which is below the current EU recommended limit. Compared to the other toxins produced by *F. verticillioides*, FB_1_ is the main toxin produced by this fungi, occurring more frequently and most toxic and, therefore, also most frequently studied (Galeana-Sánchez et al. 2017).

This study is the first to evaluate the sensitivity of rainbow trout to fumonisins. Trout exposed to the FU_mix_ (with a sum of FB_1_ and FB_2_ being 9000 µg/kg) showed a significant reduction in the growth, but only during restrictive feeding and not during *ad libitum* feeding. The sensitivity of fish to fumonisins seems to differ strongly between fish species. In studies with a longer *ad libitum* period than the current study, lower fumonisins levels resulted in reduced growth in seabream (*FB*_1_ and *FB*_2_ ≥ 168 µg/kg; (Gonçalves et al. 2020a)) and in turbot (*FB*_1_ and *FB*_2_ ≥ 1000 µg/kg; (Gonçalves et al. 2020b)). In other fish species, fumonisins effects on growth were only observed at higher levels (*FB*_1_ ≥ 5000 µg/kg in African catfish; (Gbore et al. 2010)) (*FB*_1_ ≥ 40,000 µg/kg in Nile tilapia; (Tuan et al. 2003)) (*FB*_1_ ≥ 20,000 µg/kg in channel catfish; (Lumlertdacha et al. 1995); (Yildirim et al. 2000)). The disappearance of the FU_mix_ on growth during the *ad libitum* period might suggest that trout adapted to FU_mix_ exposure. In other words, the fish may have become less sensitive to the toxic effects of this mixture. However, liver and intestinal histopathological observations do not support this hypothesis of adapting to these toxins. Instead, various histopathological measurements (e.g., increased glycogen vacuolization in the liver and reduced mucosal fold and enterocyte width in the gastrointestinal tract) revealed that FU_mix_ effects aggravated with time, being more severe at the end of the *ad libitum* feeding period. The time (or feeding level) related change in FU_mix_ effects together with the large variability between fish species in sensitivity to *F. verticillioides* toxins warrants further research on this group of toxins to improve the current recommended EU limits. The approach taken in the current study to use a mixture of *F. verticillioides* produced toxins can be advised as approach also for other fish species because feed ingredients with an infestation of *F. verticillioides* are most likely to contain a mixture of fusaric acid and FB_1_, FB_2_ and FB_3_.

The main objective of this study was to investigate the presence of interaction effects (antagonism, synergism or additivity) of FU_mix_ and DON. For growth performance data during both feeding periods (restrictive and *ad libitum*), no significant interaction effects were present (Tables 2 and 3), which suggests that the effects of FU_mix_ and DON are additive during co-exposure. Apart from the goblet cell density of the pyloric caeca, all studied histological measurements suggested additivity of FU_mix_ and DON effect. It can be hypothesised that the combination of *Fusarium* spp. toxins, as applied in the current study (FU_mix_ versus DON), does not influence each other’s toxicological effects. It has been suggested that combining mycotoxins with structural similarities, comparable modes of action and thus toxicity profiles, increases the likelihood that their effects are additive (Speijers and Speijers 2004). The absence of a significant interaction effect might also be related to low statistical power of this study (a too low number of tanks/animals being included into the study). A major toxicological impact FB_1_, the most abundant toxin produced by *F. verticillioides*, is an interference with the sphingolipids’ metabolism via inhibition of ceramide synthase enzymes (Feijó Corrêa et al. 2018), which results in an alteration of the sphinganine/sphingosine ratio in livers. Therefore, this ratio is used as a biomarker of FB_1_ exposure (Riley et al. 1994). It can also be the case that the proper measurements for quantifying FU_mix_ effects were not assessed in the present study in order to reveal interaction effects (e.g., the sphinganine/sphingosine ratio in the liver).

Only few studies in fish addressed co-exposure; thus, the comparison between effects of co-exposure to FU_mix_ and DON is only possible with terrestrial animal literature. Feeds and also ingredients are often co-contaminated with multiple toxins (Streit et al. 2012). Next to the limited information on the effects of co-occurrence, also in terrestrial animals, there is a large variability in responses between studies, species and the measured indicators (Smith et al. 2016). In pigs, an early study (Smith et al. 1997) found synergism between DON and fusaric acid on growth performance. In contrast, a later study in pigs (Grenier et al. 2011) did not show a interaction effect between DON and fumonisins on growth, but a synergistic action was observed regarding the severity of histopathological lesions in the liver. In ducks, synergism between fumonisins, DON and ZEN resulted a lower growth, but this was not observed in any of the other factors assessed (Peillod et al. 2021). In another pig study (Bracarense et al. 2012), synergism, antogonism and additivity were observed for the co-exposure to DON and fumonisins depending on the assessed measurement. Due to the large variability between and within studies, further in vitro and in vivo research is required to understand and explain the combined mycotoxin effects and to predict their interactions. Such information is needed for regulatory authorities of the animal feed industry in formulating recommended limits for mycotoxin mixtures.

This first rainbow trout study evaluating the combined effects of the most prevalent mycotoxins in aquafeeds produced by *F. graminearum*: DON and *F. verticillioides*: FU_mix_ (fusaric acid and FB_1_, FB_2_ and FB_3_) showed that the co-exposure of FU_mix_ and DON primarily had additive effects on growth performance (no interaction effects). The exposure to FU_mix_ and to DON impaired growth and FCR during the restrictive feeding period. During *ad libitum* feeding, growth and feed intake were reduced by DON exposure, but not by FU_mix_. There were no toxins interaction effects on histopathological measurements in the liver and gastrointestinal tract. DON exposure in the current study resulted in minor histological changes, and FU_mix_ did lead to minor alteration in liver and intestinal tissue but mainly at the end of the *ad libitum* feeding period.

In conclusion, despite the minor impact on the liver, the current study clearly shows a substantial effect on growth performance already at a DON exposure of level of 2700 µg/kg feed. This implies that the current EU recommended limit for DON at 5000 µg/kg may need to be reconsidered for fish. Since no other studies in trout have evaluated the effects of the sum of FB_1_ and FB_2_, a conclusion cannot be drawn about the effectiveness of the EU recommended limit at 10,000 µg/kg, although it is suggested future studies to measure the effects of FU_mix_ instead of the sum of FB_1_ and FB_2_.

## Data Availability

The corresponding author can be contacted if access to the data is desired.
